# Optic Pathway–Hypothalamic Glioma Apoplexy: A Report of Two Cases and Systematic Review of the Literature

**DOI:** 10.3389/fsurg.2022.891556

**Published:** 2022-05-30

**Authors:** Saleh Baeesa, Yazid Maghrabi, Rana Moshref, Jaudah Al-Maghrabi

**Affiliations:** ^1^ Department of Neurosciences, King Faisal Specialist Hospital and Research Center, Jeddah, Saudi Arabia; ^2^ Department of Pathology, King Faisal Specialist Hospital and Research Center, Jeddah, Saudi Arabia

**Keywords:** optic glioma, hypothalamic glioma, hemorrhage, apoplexy, optic chiasm

## Abstract

**Background:**

Hemorrhage into optic pathway–hypothalamic glioma (OPHG) is rare. Variable clinical presentations and outcomes are associated with such pathology. We aim to present two infants presented with OPHG and a systematic review of the literature.

**Methods:**

We describe two cases of infants presenting with sudden decreased vision, poor feeding, and irritability due to OPHG. Both patients underwent urgent craniotomy and subtotal resection followed by chemotherapy. We systematically reviewed the literature using PubMed, Google Scholar, and Embase. In addition, we included all English published reports for all ages discussing the optic pathway (optic nerve and optic chiasm) or hypothalamic glioma associated with hemorrhage from the year of the first reported case (1970) to January 2022.

**Results:**

Of 17,949, 44 articles met the inclusion criteria of this review. A total of 56 cases were described with a mean of 21.35 years (0.5–70), with the male gender 52% and the female gender 45%. The hemorrhage location was sellar/suprasellar in 43% cases. Histopathology of included cases was pilocytic astrocytoma in 41%, followed by pilomyxoid astrocytoma in 16% cases. The outcome was unfavorable; 37.5% cases showed improvement, whereas 18% cases resulted in death.

**Conclusion:**

Apoplexy of the OPHG can be fatal and associated with poor outcomes. A systematic review of the literature has shown that younger age, pilocytic or pilomexyoid astrocytoma histopathology, and chiasmal/hypothalamic locations are associated with a higher risk of intertumoral hemorrhage and poor prognosis. Further genetic studies for OPHG may provide information for high-risk patients.

## Introduction

Optic pathway–hypothalamic glioma (OPHG) can be found anywhere along the optic pathway, often in the chiasmatic-hypothalamic region (1). These tumors account for 3%–5% of all pediatric brain neoplasms and can also be diagnosed in adulthood (2–4). The most common histopathology encountered is World Health Organization (WHO) grade 1 pilocytic astrocytoma, followed by pilomyxoid astrocytoma (2, 3).

The initial clinical presentation would be visual disturbance with cases that progress to blindness (5). Due to the proximity of these lesions to sellar and suprasellar structures, endocrine and hypothalamic dysfunction would be observed (5). Moreover, as these lesions are also close to the cerebrospinal fluid pathway, hydrocephalus can result from their compressive effect (5). This type of pathology poses verities of challenges as it has a wide spectrum of symptomatology, and it is close to many eloquent brain structures, with treatment tailored on a case-by-case basis (6)

Intertumoral hemorrhage has always been linked to malignant tumors (6). Pituitary macroadenoma is considered the most common low-grade tumor associated with hemorrhage (7). Bleeding into optic gliomas is considered rare, and the highest rate of intertumoral hemorrhage was associated with pilocytic astrocytoma according to multiple series (8–10).

Herein, we describe two cases of infants who presented with hemorrhage into optic glioma, with a systematic review of the current literature regarding such presentation and outcome.

## Methods

### Cases’ Descriptions

We describe two cases of infants presenting with sudden decreased vision, poor feeding, and irritability due to optic chiasmatic/hypothalamic glioma. Both patients underwent urgent craniotomy and subtotal resection followed by chemotherapy. This study was approved by the Institutional Research Board (IRB # 2022-CR-06).

#### Case 1

A 9-month-old baby boy presented with his parents to the emergency department with the inability to follow objects, repeated vomiting, and irritability for three days. He is an outcome of uneventful full-term gestation. His postnatal history was unremarkable and neurodevelopmental milestones were normal.

On examination, he was dehydrated and frequently cried. His head circumference was in the upper 90 percentiles for age, but the anterior fontanel was small with no evidence of increased intracranial pressure. There were no stigmata of neurofibromatosis type 1 (NF1). The neurological examination revealed adequate power and tone in all extremities. There is a poor pupillary response to light and no clear visual recognition of moving objects. There was no ophthalmoplegia or facial weakness, and the corneal and gag reflexes were brisk.

**Figure 1:**
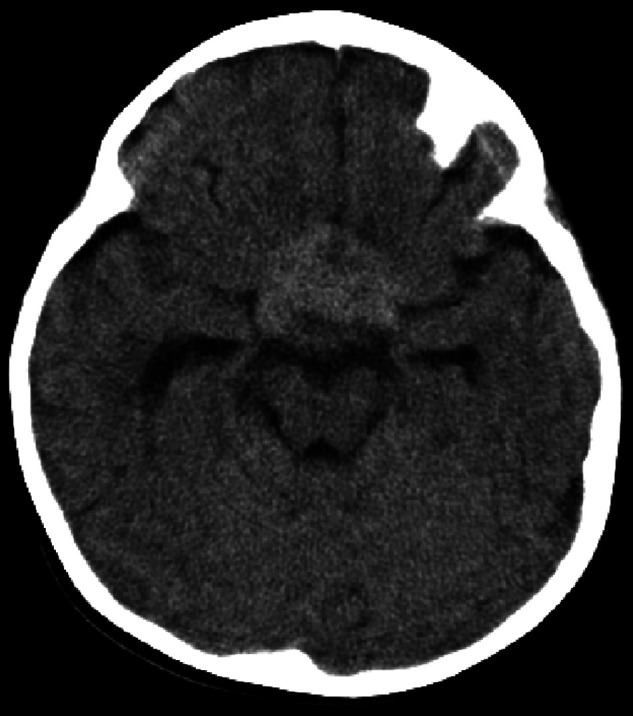
CT scan of the brain (case 1) revealed a large hyperdense suprasellar lesion and mild ventricular dilatation.

**Figure 2:**
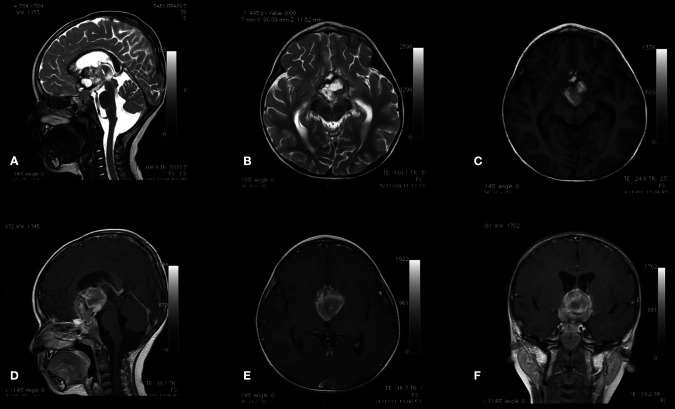
Sagittal (**A**) and axial (**B**) T2-WI and axial (**C**) T1-WI MRI scan sequences of the brain reveal large sellar and suprasellar cystic and solid lesions containing subacute components of blood products. The lesion is enhanced heterogeneously following intravenous contrast administration (**D–F**).

Urgent computed tomography (CT) scan revealed a large hyperdense suprasellar lesion and mild ventricular dilatation (Figure 1). A magnetic resonance imaging (MRI) scan revealed large sellar and suprasellar cystic and solid lesions containing subacute components of blood products. The lesion has marked heterogeneous enhancement following intravenous contrast administration, and there is marked mass effect and mild ventriculomegaly (Figure 2). Magnetic resonance angiography (MRA) revealed no vascular abnormality.

The baby had normal routine laboratory workup with particularly no coagulopathy or pituitary dysfunction on admission. He was admitted to the pediatric intensive care unit (PICU) to optimize her general condition. Ophthalmological consultation documented his poor visual response. He was started on dexamethasone (2 mg intravenous injection followed by 0.5 mg hourly). The surgery was performed the next day on the right frontotemporal craniotomy. The dura was open curvilinear and reflected anteriorly. There was no significant brain swelling, and there was no need to insert a ventricular drain. The optic-carotid cistern and sylvian fissure were widely opened, which made more brain relaxation. The tumor was yellowish firm, with areas of soft cystic consistencies. Intratumoral microscopic decompression of the suprachiasmatic and hypothalamic solid and cystic parts was achieved using an ultrasonic aspirator. There were mixed-blood products of recent and old components within cystic regions. After achieving adequate decompression, craniotomy closure was done in layers. The patient was transferred to PICU in stable condition with an estimated blood loss of around 120 ml. He received an intraoperative blood transfusion of 100 ml.

The patient had an uneventful postoperative period; he was extubated three days later. There was no postoperative endocrinopathy, and his visual status was the same. Postoperative brain MRI revealed adequate decompression of 50% of the tumor, and he was discharged in stable condition 12 days after surgery.

**Figure 3:**
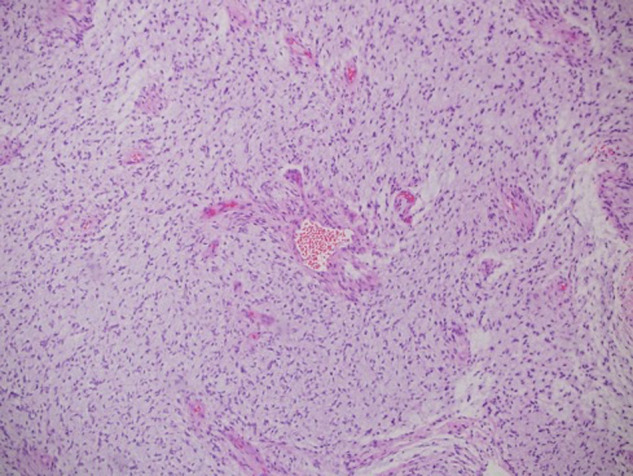
Histopathology examination showed astrocytoma with myxoid features suggestive of pilomyxoid astrocytoma with a background of hemorrhagic necrosis.

The histopathology examination was consistent with pilomyxoid astrocytoma with hemorrhagic background and necrosis (Figure 3).

**Figure 4:**
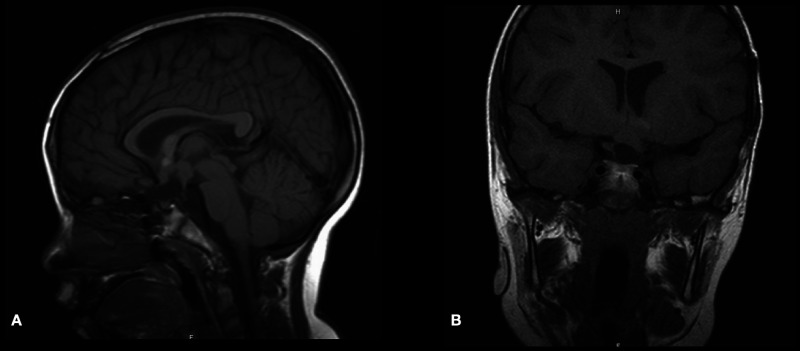
Postoperative sagittal and coronal T1-WI MRI scan demonstrating stable residual tumor at 5-year follow-up.

He was evaluated at outpatient clinics by pediatric oncology service and started on MOB chemotherapy protocol (including nitrogen mustard, vincristine, and procarbazine for one year). At the age of 5 years, his brain MRI scan revealed a good response to stable residual tumor treatment (Figure 4). His visual evaluation revealed a blind left eye, but he could see and recognize objects from the right eye.

#### Case 2

A one-year-old baby girl, with a normal state of health until two weeks from admission, presented with decreased activity and oral intake and repeated vomiting 4–5 times a day. No seizures, trauma, or loss of consciousness. She was delivered at 35-week gestation by cesarean section (CS) due to uncontrolled gestational diabetes mellitus. Her postnatal history was unremarkable and neurodevelopmental milestones were within normal.

On examination, she was awake but irritable. General physical examination revealed head circumference within the 90 percentiles for age with normal small anterior fontanel. There were multiple cafe au lait spots (more than six) in the trunk and extremities, measuring greater than 5 mm. Neurological examination revealed normal tone and power in all extremities. Her pupils were sluggish to light with decreased response to external stimuli and moving objects, but further tests could not be assessed due to the baby’s cooperation. The optical coherence tomography test was difficult to perform due to the infants’ clinical condition. However, there was no ophthalmoplegia, and her corneal response, facial movement, and gag reflex were within normal.

**Figure 5:**
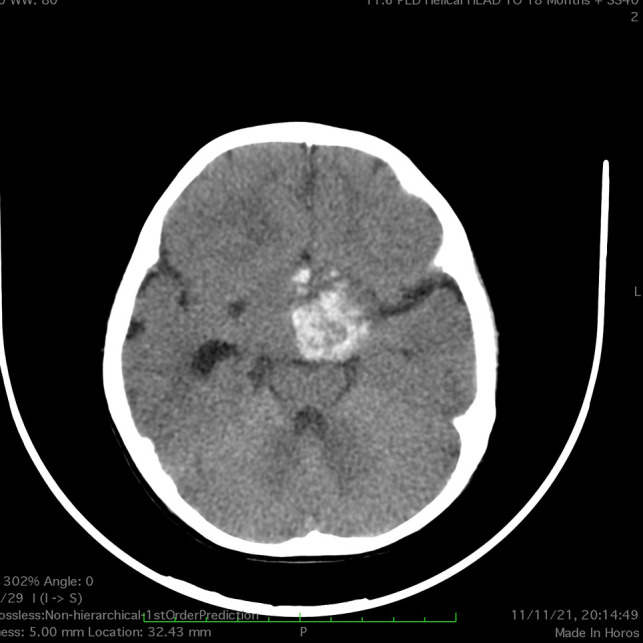
CT scan of the brain (case 2) reveals a large hyperdense suprasellar lesion and mild ventricular dilatation.

**Figure 6:**
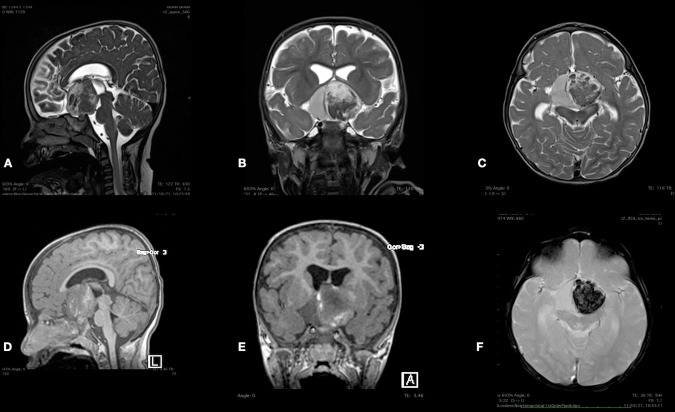
Sagittal (**A**), axial (**B**), and coronal (**C**) T2-WI and sagittal (**D**), axial (**E**) T1-WI MRI scan sequences of the brain demonstrating a large suprasellar mass with mixed solid and cystic components representing subacute hemorrhages. The T2-GRE sequence (**F**) revealed marked hypointensity of the tumor representing acute hemorrhage.

On admission, her laboratory workup was normal, with no evidence of coagulopathy. A brain CT scan was performed in the emergency department, revealing a sizeable suprasellar lesion with an internal hemorrhagic component: mild ventriculomegaly (Figure 5). A brain MRI scan demonstrated a large suprasellar mass with mixed solid and cystic components representing subacute hemorrhages, a heterogeneous enhancement following intravenous contrast administration. The ventricular systems were mildly increased in size, and MRA revealed no vascular abnormalities (Figure 6).

The patient was admitted to PICU well hydrated and started on dexamethasone (2 mg intravenous injection followed by 0.5 mg hourly). She underwent right frontotemporal craniotomy the following day. The dura was open curvilinear and reflected anteriorly. There was no significant brain swelling or edema. The sylvian fissure and optic-carotid cistern were opened, which made more brain relaxation. The tumor was yellowish soft, with areas of firm and areas of soft cystic consistencies. Intratumoral microscopic decompression of the suprachiasmatic and hypothalamic solid and cystic parts was achieved using an ultrasonic aspirator. There were old and new blood products within the large cystic component. After achieving adequate decompression, craniotomy closure was done in layers, and the patient was transferred to PICU in stable condition with an estimated blood loss of around 200 ml. She received an intraoperative blood transfusion of 150 ml.

The patient remained ventilated in PICU for two days after surgery. Her postoperative course revealed transient diabetic insipidus, and she received two doses of desmopressin for two days. Her neurological examination was unchanged. A postoperative brain MRI scan (within 24 h) revealed 60%–70% adequate decompression. The patient was evaluated by pediatric oncology and endocrinology services and discharged on Day 10. She was up to date on her neurodevelopmental milestones: able to cruise on furniture, sit without support, has a good pincer grasp in both hands, able to say 2–3 words, recognize faces and follow objects, and interact with her parents and her elder sister. Pediatric neurology discharged her on clobazam 2.5 mg at bedtime for insomnia and myoclonic seizures and levothyroxine 0.25 mg as per pediatric endocrinology advice.

**Figure 7:**
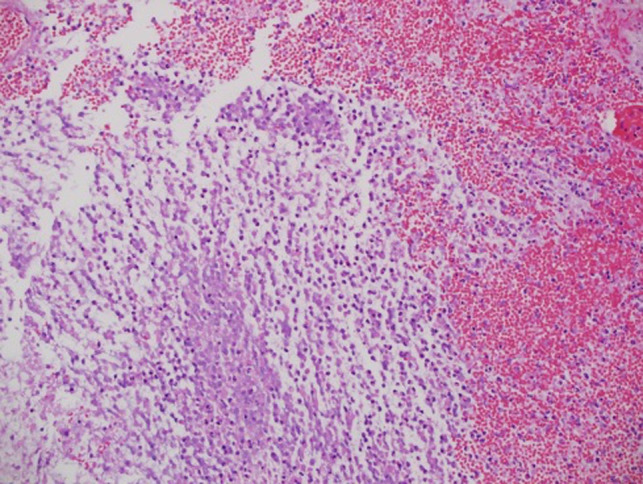
Histopathology examination of the specimen showed astrocytoma with myxoid features suggestive of pilomyxoid astrocytoma with a background of hemorrhagic necrosis.

Histopathology examination of the specimen showed astrocytoma with myxoid features suggestive of pilomyxoid astrocytoma with a background of hemorrhage and necrosis (Figure 7). Therefore, genetics was done and a WES study was recommended to rule out NF1.

**Figure 8:**
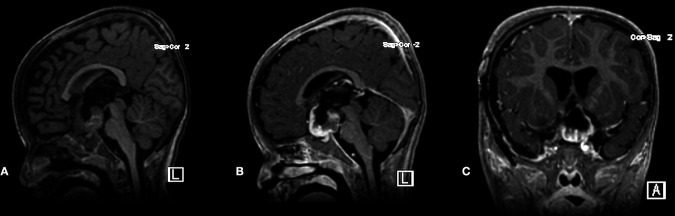
Postoperative plain sagittal T1-WI MRI scan and post-contrast T1-WI sagittal and coronal MRI scans demonstrated adequate decompression and stable residual tumor at 6-month follow-up.

A pediatric oncology service followed the patient. She was labeled NF1 as she met the following two criteria: cafe au lait and positive family history (her father and elder sister had cutaneous stigmata of NF1). The patient started on chemotherapy protocol Cog a9952, Carboplatin Plus Vincristine for one year. At six months, an early brain MRI scan revealed a good response to the treatment (Figure 8).

### Qualitative Systematic Review

This study was reported in accordance with Preferred Reporting Items for Systematic Reviews and Meta-Analyses (PRISMA) guidelines.

#### Search Strategy

An extensive literature search was conducted to include English-published case reports discussing optic pathway/hypothalamic glioma hemorrhage, covering the time from January 1970 (date of first reported case) up to January 2022. Several databases were utilized, including PubMed, Google Scholar, and Embase. Several related keywords were used, such as “optic glioma,” “chiasm,” “hypothalamus,” “hemorrhage,” “apoplexy,” and “bleeding.”

#### Inclusion Criteria

All case reports or case series of the optic pathway (optic nerve and optic chiasm) or hypothalamic glioma associated with hemorrhage for pediatric and adult populations were included from the first reported case (1970) to January 2022. Cases dealing with other hemorrhagic lesions and vascular lesions in the optic pathway/hypothalamus were excluded.

#### Data Extraction

Abstracts were reviewed by two authors (RM and YM), inclusion criteria were applied, and any disagreement was resolved by discussion and review with the senior author (SB). Full-text abstracts that met the inclusion criteria were accessed and reviewed. For all included cases, the following variables were collected: author/year, age in years, gender, history of neurofibromatosis type 1 (NF1), pertinent medical history, clinical features, location of the hemorrhage, treatment received, histological diagnosis, and outcome. All variables were collected using a Microsoft Excel Sheet.

## Results

**Figure 9:**
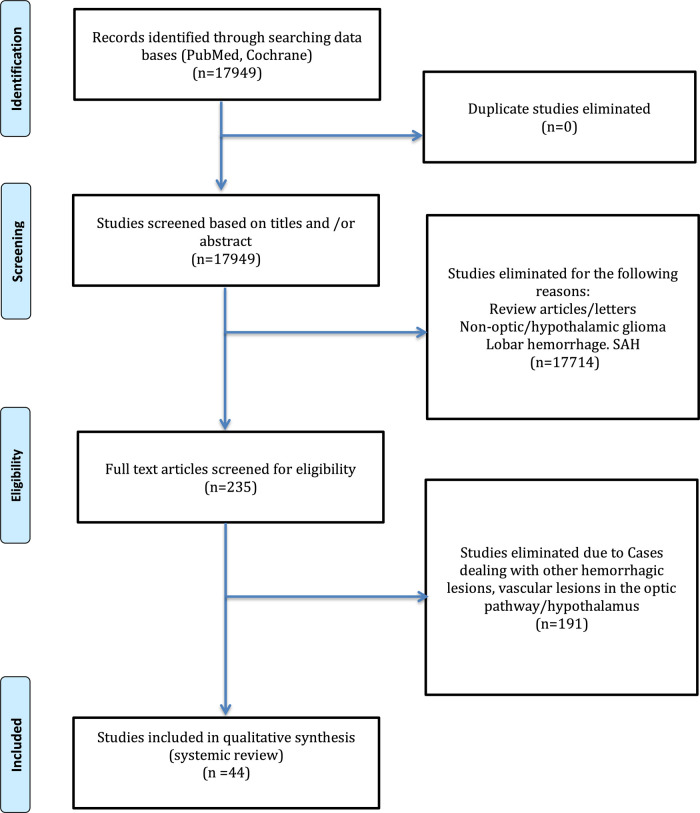
PRISMA flowchart of the review process.

Our search strategy yielded 17,949 articles, out of which 235 were included for full-text review. Only 44 articles were included in the final qualitative analysis (Figure 9). The summary of included articles of the 56 cases is shown in Table 1. All included articles were of low level of evidence as the majority were either case reports or case series. There were around 56 optic pathway/hypothalamic glioma-associated hemorrhage cases from the included articles, including our reported cases.

**Table 1:** Summary of included studies.

No.	Author (R)	Age (Y)	Gender	Presentation	NF	Visual acuity	Hemorrhage location	Hydrocephalus	Treatment	Histopathology	Outcome
1	Current cases	0.75	M	No	No	Decreased	Sellar/suprasellar	No	Partial resection, CTx	Pilomyxoid astrocytoma	Blind left eye, stable residual at 5 years F/U
2		1	F	No	Yes	Decreased	Suprasellar	No	Partial resection, CTx	Pilomyxoid astrocytoma	Improvement at 6 months F/U
3	Din et al. (11)	15	F	NR	NR	NR	Sellar/suprasellar	NR	RTx	Gliosarcoma	Died
4	Ishi et al. (12)	4	F	NR	NR	NR	IVH	NR	Partial resection, CTx, RTx	Pilomyxoid astrocytoma (FGFR1 K656E)	Died
5		6	M	N&V	NR	NR	Suprasellar	Yes	Tumor resection, CTx, RTx	Pilomyxoid astrocytoma (FGFR1 D652G)	No recurrence at 11 years F/U
6		25	M	Coma	NR	NR	Suprasellar + IVH	Yes	Ventricular drainage	Pilocytic Astrocytoma (FGFR1 N546K)	Comatose
7	Lu and Xu (13)	10	M	NR	NR	Blindness	Rt optic disc/nerve	NR	Tumor resection	Astrocytoma	Improvement
8	Campbell et al. (14)	1.6	M	Proptosis	NR	NR	Rt optic disc	NR	Tumor biopsy	Pilocytic astrocytoma, BRAF positive	NR
9	Cortez et al. (15)	55	M	Headache, dizziness	No	NR	Suprasellar	NR	Tumor resection	Pilocytic astrocytoma, BRAF negative	Right hemiparesis, improvement of dysphagia
10	Baarsen et al. (6)	7	M	Decreased consciousness	No	Decreased	Suprasellar, frontal, IVH	Yes	Partial resection, EVD	Pilocytic astrocytoma BRAF −ve	Improvement
11		13	M	H, dizziness, fear attacks	Yes	NR	Chiasm, rectus gyri	NR	Low dose steroids, antipsychotics	Low-grade astrocytoma	Improvement
12		30	F	H, decreased consciousness	No	NR	Suprasellar, hypothalamic	Yes	Hematoma evacuation, partial tumor resection	Low-grade astrocytoma	Gradual improvement
13		15	M	H, decreased consciousness	No	NR	Chiasm, IVH	Yes	Observation, VP shunt	Pilocytic Astrocytoma	Died
14		4	M	H, decreased consciousness	No	NR	IVH	Yes	Tumor biopsy, VP shunt	Pilocytic/pilomyxoid astrocytoma, BRAF positive, leptomeningeal metastases	Improvement and stable on MEK inhibitors
15		15	F	H, decreased consciousness	No	Decreased	Chiasm	Yes	hematoma evacuation, tumor biopsy	Low-grade glioma	No improvement
16		22	F	H, decreased consciousness	No	Decreased	Chiasm	Yes	hematoma evacuation, partial tumor resection	Low-grade glioma	Improvement in consciousness, Rt eye blind
17		6	F	H, V, decreased consciousness, Lt CN VII palsy	No	NR	Chiasm, hypothalamus	Yes	hematoma evacuation, tumor resection	Pilomyxoid astrocytoma (Ki-67 20%)	Improvement
18		9	F	Decreased Consciousness	No	NR	Suprasellar, IVH	Yes	EVD, tumor debulking	Ganglioglioma	Improvement
19	Motoyama et al. (16)	17	M	Ocular pain	Yes	NR	Lt intraorbital/optic nerve	NR	NR	Pilocytic Astrocytoma	NR
20	Dewan et al. (17)	20	M	Ocular pain	No	Decreased	Intraorbital	NR	Left enucleation	Ependymoma grade II	Improvement
21	Mathew et al. (18)	50	M	Proptosis, ocular pain	No	NR	Intraorbital	NR	Tumor excision and debridement	Astrocytoma grade II and myiasis	Improvement
22	Serova et al. (19)	17	NR	H, V, decreased consciousness	NR	Decreased	Frontal	Yes	Partial resection	Pilomyxoid astrocytoma (Ki-67 8%)	Visual functions preserved at the preoperative level after surgery
23		22	NR	Decreased consciousness	NR	Decreased Rt	Chiasmal-sellar	Yes	Partial resection, RTx	Pilomyxoid astrocytoma (Ki-67 < 10%)	NR
24	Wang et al. (20)	13	M	H, V, coma	NR	Decreased	Suprasellar	Yes	EVD, VP shunt, GKRS, subtotal resection	Pilomyxoid astrocytoma	Improve neuro, endocrinopathy, and DI persists, no recurrence in 10 months F/U
25	Kapoor et al. (21)	8	F	H, V, VI palsy	NR	NR	Suprasellar + IVH	Yes	VP shunt, subtotal resection	Pilocytic Astrocytoma	Improvement
26	Arrese et al. (22)	30	F	Headache	No	NR	SAH	NR	Hematoma evacuation, tumor biopsy	Pilocytic astrocytoma	Improvement
27	Della puppa et al. (23)	42	F	Headache	NR	Rt decreased	Rt optic nerve	No	Subtotal resection	Pilocytic astrocytoma	Improvement of visual parameters
28	Ashur-Fabian et al. (24)	64	M	NR	No	Blindness	Rt optic nerve	NR	Tumor biopsy, CTx, RTx	Glioblastoma grade IV, Ki-67 20%	Died
29	Faraji et al. (25)	45	F	Headache, confusion	No	NR	Hypothalamic, SAH	NR	Tumor biopsy, EVD, CTx, RTx	Astrocytoma grade II Ki-67 5%	Improvement
30	Liu et al. (26)	70	M	Ocular pain	No	Decreased	Lt optic nerve	NR	Steroid, tumor resection	Astrocytoma grade III	Died
31	Ball et al. (27)	5	F	Vomiting, decreased consciousness	No	NR	IVH	NR	Hematoma evacuation, tumor biopsy, EVD	Astrocytoma grade III	Improvement
32	Hill et al. (5)	17	F	Headache, vomiting	No	NR	IVH	NR	Tumor biopsy	Pilocytic astrocytoma	Improvement
33	Vogel (28)	16	F	Dizziness, headache, meningismus	No	NR	Chiasm	NR	ICA aneurysm embolization	Pilocytic astrocytoma	Unchanged
34	Shibahara et al. (10)	18	M	Decreased consciousness	NR	NR	SAH, IVH	NR	Observation	Pilomyxoid astrocytoma	NR
35	Hamada et al. (29)	5	M	Headache, vomiting	NR	NR	IVH	NR	Hematoma evacuation, partial tumor resection	Pilomyxoid astrocytoma	Died
36	White et al. (30)	12	F	Headache	NR	NR	Hypothalamic	NR	Tumor biopsy	Pilomyxoid astrocytoma	NR
37	Garg et al. (31)	13	M	Headache, vomiting, decreased consciousness	NR	YES	Subarachnoidal, interventricular	NR	Tumor resection	Pilocytic astrocytoma	Improvement
38	Yokoyama et al. (32)	33	F	NR	No	Blindness	Lt optic nerve and chiasm	NR	Cesarean section, hematoma evacuation, tumor biopsy	Fibrillary astrocytoma	Improvement
39	Aichholzer et al. (33)	11	M	Vomiting, decreased consciousness	No	Blind right eye	Suprasellar, SAH, IVH	NR	Clipping of tumor encased ACoA aneurysm	Pilocytic astrocytoma	Died
40	Devi et al. (34)	4	M	Vomiting, decreased consciousness, dilated pupils	No	NR	SAH, IVH	NR	EVD, steroids, ventilation	Pilocytic astrocytoma	Died
41	Wright et al. (35)	70	M	NR	No	Bilateral visual loss	Chiasm	No	Stereotactic biopsy	GBM	Died
42	Golash et al. (36)	13	F	Pain, decreased vision, vomiting, diplopia	No	NR	Frontal basel ICH	NR	VP shunt, tumor biopsy	Pilocytic astrocytoma	Improvement
43	Hwang et al. (37)	34	M	Decreased consciousness	NR	NR	Hypothalamic	NR	Tumor resection	Pilocytic astrocytoma	Improvement
44	Matsumoto et al. (38)	45	M	Headache	NR	NR	SAH, IVH	NR	Hematoma evacuation, tumor resection	Pilocytic astrocytoma	Improvement
45	Hasegawa et al. (39)	54	F	Headache, decreased vision, gait disturbance	No	NR	Hypothalamic	NR	Subtotal tumor resection, VP shunt, RTx	Astrocytoma GFAP +ve, cavernous angioma	Died
46	Sorenson et al. (40)	58	F	Transient memory loss	No	NR	Hypothalamic	NR	Tumor biopsy	Pilocytic astrocytoma	Improvement
47	Byard et al. (41)	5	F	NR	NR	NR	IVH	NR	NR	Astrocytoma	NR
48	Applegate and Pribram (42)	16	F	Proptosis	Yes	NR	Intraorbital	NR	Observation	Pilocytic astrocytoma	Improvement
49	Jordan et al. (43)	27	M	Proptosis	No	Blindness	Intraorbital	NR	Tumor resection and hematoma evacuation	Pilocytic astrocytoma	Improvement
50	Yokota et al. (44)	7	M	Headache, vomiting	NR	NR	Hypothalamic	NR	Tumor excision	Astrocytoma grade III	Died
51	Maitland et al. (45)	15	F	NR	NR	Decreased	Suprasellar	NR	Hematoma evacuation, tumor biopsy	Low-grade astrocytoma	Visual improvement
52	Waga et al. (46)	0.5	M	NR	No	NR	Suprasellar, chiasm, optic nerves	NR	Subtotal resection of the tumor, RTx	Astrocytoma grade III	Diabetes insipidus, hyponatremia
53	Charles et al. (47)	26	F	Proptosis	No	Blindness	Intraorbital	NR	Hematoma and optic nerve removal	Pilocytic astrocytoma	NR
54	Yanoff and Zimmerman (48)	14	M	Proptosis	NR	Blindness	Intraorbital	NR	Enucleation	Pilocytic astrocytoma	NR
55	Glew (49)	30	M	Headache decreased consciousness	NR	NR	Hypothalamic	NR	Hematoma evacuation, tumor biopsy	Fibrillary astrocytoma grade I	Died
56	Schiender et al. (50)	10	F	Headache, vomiting, proptosis	Yes	NR	The optic nerve, chiasm	NR	Hematoma evacuation, tumor biopsy	Pilocytic astrocytoma	Improvement

*NA, not applicable; CTx, chemotherapy; F/U, follow up; NR, not reported; RTx, radiotherapy; NF, neurofiromatosis; IVH: intraventricular hemorrhage; N, nausea; V, vomiting; Rt, right; Lt, left; HTN, hypertension; H, headache; SAH, subarachnoid hemorrhage; GKRS, gamma knife radiosurgery; VP, ventriculoperitoneal shunt; EVD, external ventricular drain*; *GFAP, glial fibrillary acidic protein; GBM, glioblastoma multiforme; ACoA, anterior communicating artery*.

The mean age of included case was 21.35 years (0.5–70), with the male gender comprising 52% and the female gender comprising 45%. The presence of neurofibromatosis stigmata was mentioned in five cases (9%), absent in 52%, and not reported in 39.3% cases.

Clinically, in 37.5% cases, patients experienced decreased visual acuity and even vision loss, while 62.5% failed to mention that. Visual field deficits were reported in only 11 cases (20%), while it was not possible to examine due to the young age of the patients in 9% cases. Some patients, especially those with sellar/suprasellar hemorrhage, presented with high ICP symptoms (29%).

The hemorrhage location was sellar/suprasellar in 43% cases, followed by intraventricular hemorrhage in 20%. In 14% cases, hemorrhage was confined to the orbit.

Histopathology of included cases was pilocytic astrocytoma in 41% cases, pilomyxoid astrocytoma in 16% cases, followed by others (43%), including cases of glioblastoma, gliosarcoma, high-grade diffuse astrocytoma, and ganglioglioma.

Regarding outcome, 37.5% cases showed improvement, whereas 18% resulted in death. Three were highly associated with highly malignant types of gliomas (glioblastoma and gliosarcoma). Others were related to the chiasmal/hypothalamic hemorrhage location. It has also been observed that in chiasmatic/hypothalamic glioma cases, the persistence of multiple endocrinopathies and diabetes insipidus in 4% patients.

## Discussion

It has been discussed in the literature that intertumoral hemorrhage into optic pathway/hypothalamic glioma is rare (6). Our cases above add to the reported cases of such rare occurrences.

In regard to risk factors of intertumoral hemorrhage into gliomas, it has been speculated that a young age can be associated with a higher risk of hemorrhage (7, 10, 47). Although low-grade gliomas such as pilocytic astrocytoma are frequent in the younger population, some might argue that intertumoral hemorrhage in optic gliomas is rather related to tumor histopathology than age (6). Even though reported cases did not show any meaningful gender difference, pregnancy would be one of the risk factors that lead to the development of intertumoral hemorrhage, as hypothesized by Czyzyk and associates (51).

From our systematic review and with the inclusion of our own described cases, we found that the histopathology of 42.9% of included cases was pilocytic astrocytoma, followed by pilomyxoid astrocytoma. This would support the hypothesis that these tumors have a higher risk of associated hemorrhage, as these tumors might be more vascular with vessel proliferation and thinner vessel walls (21, 29). In addition, Ishi and colleagues have speculated that low-grade gliomas with FGFR1 mutation are associated with spontaneous hemorrhage in adult and pediatric populations, as evidenced by their retrospective review of 66 patients (12).

Concerning NF stigmata, most of the cases did not mention the absence of such occurrence. Shibahara et al. argued that NF1 is associated with a higher rate of intertumoral hemorrhage in gliomas (10). However, still not enough evidence supporting this hypothesis.

Clinical presentation varies between cases and depending on the age, as evident in the included cases. Most of the symptoms and signs are related to either visual deterioration or elevated intracranial pressure. Furthermore, patients may complain of decreased visual acuity in the affected eye/s and visual field cut depending on the site of hemorrhage and proptosis (Table 1).

As these hemorrhages can present with signs of high ICP requiring urgent intervention, a CT scan is the modality of choice to determine the site and extent of hemorrhage (6). On the other hand, MRI is useful for differentiating between optic pathway tumors, pituitary tumors, and vascular anomalies (32). Vascular imaging is required in cases of bleeding into the optic pathway as it can result from cavernous or arteriovenous malformations in such locations (52, 53).

Our systematic review has shown how variable treatment options for such pathology range from observation to extensive tumor resection. Most cases have been managed by evacuating the resultant hematoma with decompression of the tumor. However, some reports have carried enucleation of the affected optic nerve, as reported by Yanoff et al. and Dawan et al. (17, 48).

Ten deaths have been reported in the included literature, most of which were related to intraventricular hemorrhage or hemorrhage into the hypothalamus. Out of these 10 deaths, three cases were due to high-grade gliomas (glioblastoma and gliosarcoma) (11, 24, 35). On the other hand, van Baarsen and colleagues suggested that hemorrhage into glioma of the optic nerves carries a more benign course than hypothalamic and chiasmal ones since this type of hemorrhage is confined to the orbit and does not cause intracerebral damage (6).

As mentioned earlier, management decisions should be tailored based on the case since, in some cases, surgical intervention could lead to more deterioration (6). In cases of hydrocephalus, cerebrospinal fluid diversion needs to be done on an urgent basis (6). In other instances, disturbance of the level of consciousness might be due to a compressive effect on the hypothalamus, where surgical decompression and hematoma evacuation are indicated (6). In other cases, surgery is indicated to prevent further deterioration of vision and for histopathological diagnosis (6).

## Conclusion

Intertumoral hemorrhage into optic pathway/hypothalamic gliomas is a rare occurrence. We described two infants presenting with sudden decreased vision, poor feeding, and irritability. Our systematic review has shown that intratumoral hemorrhage in OPHG was prevalent at a younger age, pilocytic or pilomexyoid astrocytoma histopathology, and associated with poor prognosis. Further studies with genetic analysis of OPHG may help identify the population at a higher risk of developing such a devastating hemorrhagic complication.

## Data Availability

The raw data supporting the conclusions of this article will be made available by the authors without undue reservation.
